# Clinical Application of Just-in-Time Training to Support Resident Learning and Success in Neonatal Intubation

**DOI:** 10.7759/cureus.107923

**Published:** 2026-04-28

**Authors:** Andrew Delle Donne, Andrea F McGlynn

**Affiliations:** 1 Neonatology, Jersey Shore University Medical Center, Neptune, USA; 2 Statistics, Naval Medical Center Portsmouth, Portsmouth, USA

**Keywords:** graduate medical education, just-in-time training, military medicine, neonatal intubation, patient safety, patient safety-based medical education, procedural skills training, simulation in medical education

## Abstract

Background: Neonatal intubation (NI) requires technical skill and can cause life-threatening complications when performed improperly. Trainee success at NI is substandard, and nearly half of graduating trainees do not achieve the desired competency by the end of residency. This issue will likely worsen with the new ACGME rules that allow fewer intensive care rotations. The study objective was to evaluate if structured just-time-time-training (JITT) utilizing a high-fidelity neonatal mannequin was feasible and effective in a clinical environment and could improve trainee NI success. We hypothesized that JITT could be safely introduced in a clinical environment and that it would increase NI success overall, including more first-attempt success, decreased number of attempts and time-to-success, and decreased adverse outcomes.

Methods: Pediatric trainees who met the inclusion criteria and were in the neonatal intensive care unit during 2019-2020 were randomly assigned to a JITT or control group prior to performing an actual intubation. The JITT group received structured instruction on a neonatal mannequin and then simulated NI with immediate corrective feedback, whereas the control group did not receive any additional training.

Results: A total of 31 intubation encounters were evaluated, involving 18 residents (JITT: n=16, 51.6%; control: n=15, 48.4%). The mean procedure time for successful intubation was significantly lower in the JITT group (117±66 seconds) compared to the control group (194±79 seconds; *p*=.029). The occurrence of adverse outcomes was significantly reduced in the JITT group (n=12, 75%) compared to the control group (n=15,100%; *p*=.045). While improvements were noted in the JITT group for overall success (12 (75%) vs. 8 (53%)) and first-attempt success (6 (37.5%) vs. 1 (6.7%)), these differences did not reach statistical significance (both p>.05). This improvement was upheld when only trainee first enrollments were evaluated.

Conclusion: This pilot study shows that targeted skills intervention via JITT is both feasible in a clinical environment and that it does improve trainee success. This concept deserves continued attention and would benefit from larger and multi-site studies to provide adequate powering to further evaluate success and secondary metrics such as time to success and occurrence of complications.

## Introduction

Endotracheal intubation is a critical, life-saving procedure that poses significant technical challenges to both learners and educators [1]. Neonatal intubation (NI) presents further challenges, requiring unique techniques due to the smaller and more anterior airway of a newborn compared to that of older children and adults [2]. Although a relatively infrequent procedure, NI demands a high level of technical proficiency and carries the risk of life-threatening complications if performed improperly [3-6]. For extremely low birth weight infants, a successful first attempt at NI is linked to improved developmental outcomes [7]. Due to these risks, residents are often excluded from attempting intubation on younger, more fragile infants. Consequently, residents have limited opportunities to practice this procedure, both because of its overall infrequency and the careful selection of infants deemed stable enough to be intubated by a learner [8,9].

Despite the critical importance of NI skills, studies confirm that the success rate for first-year trainees remains as low as 20% [10]. Although this rate improves throughout training [11,12], as many as 50% of graduating trainees still do not achieve NI competency, which is defined as an expected success rate of at least 75% on all attempts [13,14]. The current trainee success rate demonstrates a failure to meet the educational burden and demands the implementation of a better training paradigm. Developing competent and confident physicians is particularly important for the military, as newly graduated pediatricians may be required to practice alone in geographically remote and isolated locations where neonatal intensive care support is hours away. While military physicians are taught to be prepared for practice “on an island,” it is the duty of educators to constantly strive for improved methods to create competent and confident physicians [15]. This challenge will become both more important and more difficult as residents spend less time in critical care units, including in the neonatal intensive care unit (NICU) [16].

Simulation is a valuable and commonly used tool for teaching pediatric procedural skills, including the Neonatal Resuscitation Program (NRP), especially for novice learners. While this approach is initially effective, multiple studies show that these skills and knowledge diminish over time without ongoing reinforcement [11,12,17,18]. To enhance procedural skills training and promote safety, additional adjuncts such as checklists and validated scoring tools have proven effective [19-22]. Furthermore, neonatal simulators have been extensively studied as skill-trainers and are validated as effective training devices [23,24]. Despite these generally accepted benefits, some studies argue that simulation training by itself is not enough to make a significant difference, particularly if not continuously re-taught [25-27]. The standard graduate medical education training paradigm typically includes an orientation or skills “boot camp” in addition to formal NRP training. Just-in-time-training (JITT), a process originally developed in industry, is also applicable to healthcare, as both fields share the goal of producing a high-quality outcome. Although many studies have evaluated forms of JITT in healthcare, none have examined the use of JITT simulation training coupled with immediate, real-world patient encounters in the NICU [28-31].

Although it is logical to assume that a "practice round" would improve performance on an actual NI, this study aims to demonstrate the clinical feasibility and measurable positive outcomes that result from the safe and practical implementation of JITT in a clinical setting. While the study was initially powered to evaluate procedural success, it became evident during statistical design that achieving a statistically significant sample size was not feasible within the clinical time frame. Consequently, the primary objective was amended to assess the clinical feasibility of implementing JITT for pediatric trainees in a real-world intubation encounter. Secondary objectives remained focused on evaluating efficacy, including procedural success rates, time to success, and safety profiles. We hypothesized that JITT could be safely introduced in a clinical environment and that it would increase NI success overall, including more first-attempt success, decreased number of attempts and time-to-success, and decreased adverse outcomes. 

## Materials and methods

Study design

We performed a randomized controlled educational intervention at Naval Medical Center Portsmouth (NMCP), Virginia between October 2019 and September 2020. The study protocol was reviewed and approved by NMCP’s institutional review board (IRB). NMCP is a large military treatment facility that prepares trainees for potential assignment around the globe for solo practice or practice in geographically remote areas (e.g., US Naval Hospitals Guantanamo Bay and Guam). NMCP has a level III NICU that serves as a referral center for military bases in the region.

Inclusion and exclusion criteria

All pediatric trainees rotating in the NICU were considered eligible to participate. Trainees provided informed, written consent for participation prior to randomization into a treatment group. Trainees were allowed to participate as often as they were available, with the intention of crossing over into the opposite group for subsequent enrollments. Due to some residents participating more than twice, analysis was performed on both the full data set (31 intubations) and on the first-enrollment data (18 intubations).

Intubation procedures were considered eligible for the study if the infant corrected to at least 28 weeks and weighed at least 1,000 grams. In addition, the attending physician attested that the infant was both stable and appropriate for a resident, and that there was sufficient time for randomization of the resident. Therefore, all intubations were non-emergent (e.g., elective in-and-out surfactant) and occurred in the NICU. Any infant outside the age, weight, or stability requirement was excluded, as were infants expected to have an abnormal or difficult airway.

Intervention

After identification of an intubation that met the inclusion criteria, the attending provider selected the trainee to perform the procedure and subsequently notified research personnel. Therefore, the decision of when and why to intubate was left solely to the clinical discretion of the attending physician. Similarly, decisions about pre-medication type and dose were also left up to individual discretion. To prevent bias in favor of choosing to intubate or allow the resident additional attempts, study personnel could not simultaneously serve as a researcher and attending physician. To increase observer reliability and standardize the intervention, a single NICU attending provided the educational feedback and collected all data. The JITT session occurred in an immediately adjacent, transitional nursery. Regardless of the randomization group, the resident was removed from the NICU for 5-10 minutes. Randomization of the group assignments was done based on random selection of an opaque, manila envelope designating “JITT” or “control.” To ensure that the clinical attending was blinded to the intervention group, both randomization and intervention took place outside of the NICU (attached transitional care nursery) for the prescribed intervention period. 

Trainees randomized to the intervention group received targeted intubation training based on a published skills checklist for intubation [22]. The targeted skills checklist and subsequent simulation included practical considerations such as how to position the infant, how to hold the laryngoscope and correctly insert the blade, proper adjustment to view the cords, and technique to advance the endotracheal tube (ETT). Immediate feedback was given for corrective action as needed. Immediately following JITT, the resident performed the actual intubation. The simulation mannequin utilized was Newborn Anne™, manufactured by Laerdal Medical®. This device was selected based on the work of Sawyer et al., which objectively evaluated multiple commercially available products for their ability to realistically simulate intubation [24]. 

Data collection

Demographic information collected on the infant included gestational age, weight, and indication for intubation. Information collected on the resident included their training year, training program and previous success intubating a neonate. Information about the procedure included the conduct of the procedure (pre-medication), performance of appropriate time-out, and attending physician was also collected. 

The primary clinical outcome was overall intubation success defined as placement of the ETT in the airway. Secondary outcomes were the number of attempts needed for success, time per attempt, and adverse outcomes. Each attempt started when the laryngoscope blade crossed the plane of the mouth and ended when it was removed or the attending said to stop. Adverse outcomes were defined as desaturation less than 80%, bradycardia less than 100 beats per minute, or need for bag-mask-ventilation after the attempt. Positive pressure ventilation through an appropriately placed ETT following the procedure was not considered an adverse outcome. 

No personally identifiable information was collected about the trainee or the infant. A sequential number was assigned to residents after the consent process, which was used to link an encounter to a trainee. Trainees provided signed, informed consent prior to participation. A health insurance protection and portability act (HIPAA) waiver was approved by the IRB for infants involved in the study as they were not the intended area of research. The IRB agreed that the study promoted patient safety by providing additional training for a procedure already occurring at our teaching hospital. Signage was clearly posted at all parent entrances to the NICU to appropriately notify parents of the study. 

Statistical analysis

Statistical analysis was performed by an independent biostatistician employed by NMCP. The t-test was used for comparisons involving the total time, while Fisher’s exact or Mann-Whitney test was used to test non-parametric data tests. Odds ratios and relative risk values with confidence intervals were calculated where appropriate. Analysis was performed at the 95th level of confidence. All calculations were performed using Stata Statistical Software (Release 18, College Station, TX: StataCorp LLC, 2023). 

This is a pilot study intended to power enrollment to support the primary outcome. A sample size of over 200 intubations would have been required in order to reach 80% power, assuming a 10% difference. Since it was not possible to recruit the ideal number at this single institution, the study utilized a convenience sample comprising all available subjects within the specified time period. Data on intubations that were eligible but unable to be randomized based on investigator limitations were retrospectively reviewed to determine a non-experimental success rate. For these intubations, only overall success and training level were recorded. To control for the unexpected number of trainees who participated two or more times, a secondary analysis of data looking at their first randomization was also performed. 

## Results

**Figure 1:**
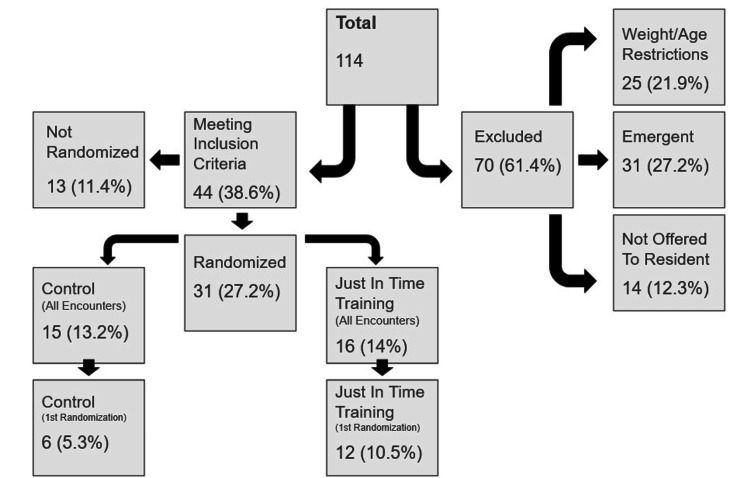
Inclusion and exclusion flowsheet for randomized intubation encounters between October 2019 and September 2020 in the Naval Medical Center Portsmouth NICU. Image created by the authors using Microsoft PowerPoint

During the study period, a total of 114 intubations occurred in the NICU, delivery room, or operating room. A total of 21.9% (n=25) of intubations were excluded due to age and or weight restrictions and another 27.2% (n=31) due to emergent status. Additionally, 12.3% (n=14) of infants were primarily intubated by an advanced practice neonatal nurse or an attending physician as part of their required skills maintenance. No infants were excluded due to the expectation of a difficult airway. A total of 38.6% (n=44) intubations were ultimately eligible for consideration, and of those, 27.2% (n=31) were successfully randomized and performed by 18 individual trainees (Figure 1). This demonstrated that this concept is indeed feasible in a clinical environment, as we were able to successfully randomize 70.4% (31/44) of eligible encounters and a significant number of the total intubations (44, 38.6%) met our strict criteria. 

The intubations not included occurred when the researcher was not notified, unavailable, or had a conflict of interest (was the attending). The JITT group and control group had 16 (14%) and 15 (13.1%) intubations per group in the initial analysis and 12 (10.5%) and 6 (5.3%) during secondary analysis of primary randomizations. Our randomization strategy was successful (Table 1). 

**Table 1:** Demographics of infants and trainees by enrollment group Statistical significance was determined at *p* <0.05. * denotes t-test ** denotes Fisher’s exact test

		Control (n=15)	Just In Timing Training (n=16)	p
Infant	Gestational Age, mean (SD)	35.0 (3.2)	35.1 (3.0)	.963*
	Weight (grams), mean (SD)	2562.0 (879.6)	2473.8 (738.6)	.128*
	Gender (male), n %	8 (53.5%)	11 (68.8%)	.437**
Trainee Year	Post-Graduate Year 1. n %	4 (26.6%)	7 (43.8%)	.732**
	Post-Graduate Year 2. n %	6 (40%)	5 (31.3%)	
	Post-Graduate Year 3. n %	5 (33.3%)	4 (25%)	

Average gestational ages and weights were nearly identical in each group. 

The JITT group demonstrated an overall success rate of 75% (n=12) compared to 53.3% (n=8) in the control group (*p*=.321). First-attempt success was also higher in the JITT group at 37.5% (n=6) versus 6.7% (n=1) in the control group (*p*=.201). While the number of attempts required for success was lower in the JITT group for both successful intubations and all encounters, these differences did not reach statistical significance (*p*=.184 and *p*=.089, respectively; Table 2).

**Table 2:** Results of trainee success and infant adverse outcomes (AE) Statistical significance was determined at *p* <0.05. * denotes t-test ** denotes Fisher’s exact test + denotes the Mann-Whitney test

		Control (n=15)	Just In Time Training ( n=16)	p
Learner Outcomes	Overall Success, n %	8 (53.3%)	12 (75%)	.273**
	First Attempt Success, n %	1 (6.7%)	6 (37.5%)	.083**
	Number of Attempts Overall, mean (SD)	2.6 (0.83)	2.1 (1.09)	.184+
	Number of Attempts (If/When Successful) (seconds), mean (SD)	2.6 (0.92)	1.8 (1.03)	.098+
	Procedure Time Total (seconds), mean (SD)	183.9 (66.9)	122.7 (60)	0.012*
	Total Procedure Time (If/When Successful) (seconds), mean (SD)	194 (79.2)	117.2 (66)	0.029*
Infant Outcomes	Desaturation, n %	12 (80%)	9 (56.3%)	.252**
	Bradycardia, n %	6 (40%)	2 (12.5%)	.113**
	Positive Pressure Ventilation, n %	9 (60%)	4 (31.3%)	.156**
	0 Total Adverse Events, n %	0 (0%)	4 (25%)	.045**
	1 Total Adverse Events, n %	7 (46.7%)	8 (50%)	
	2 Total Adverse Events, n %	4 (26.7%)	4 (25%)	
	3 Total Adverse Events, n %	4 (26.7%)	0 (0%)	

In contrast, procedure time was significantly reduced with the JITT intervention. For all intubation encounters, the mean time was 122.7 seconds in the JITT group versus 183.9 seconds in the control group (*p*=.012). For successful encounters specifically, the JITT group was significantly faster than the control group, with a mean time of 117.2 seconds versus 194.1 seconds (*p*=.029; Table 2).

The occurrence of each individual adverse outcome was lower in the JITT group; however, none of these specific metrics reached statistical significance. While the frequency of any single adverse outcome did not differ statistically, the total number of adverse outcomes per encounter was significantly higher in the control group compared to the JITT group (*p*=.045; Table 2).

Secondary analysis of first-enrollment attempts yielded similar trends. Success in the JITT group was 66.7% (8/12) versus 33.3% (2/6) in the control group. The relative risk of success for JITT versus control was 2.0 (95% CI: 0.60-6.64). Furthermore, the estimated odds of success for intubation following JITT were four times those of the control group (OR: 4.0; 95% CI: 0.50-31.98). While these secondary findings did not reach statistical significance, likely due to the limited sample size, they provide a strong signal for a larger future study (Table 3).

**Table 3:** Results of intubation success (trainee first-randomization only) ** denotes Fisher’s exact test Statistical significance was determined at *p *<0.05.

Intubation Type	Successful Intubations	Unsuccessful Intubations	p
First Randomizations Number, n %	10 (55.6%)	8 (44.4%)	
Just In Time Training, n %	8 (66.7%)	4 (33.3%)	.321**
Control, n %	2 (33.3%)	4 (66.7%)	

Study results were also compared to a separate cohort of intubations that met inclusion criteria but were not randomized; this served as an additional control measure to establish baseline resident success. The success rate for these eligible, non-randomized intubations was 53.8% (7/13), which was nearly identical to the 53.3% success rate (8/15) observed in the randomized control group. Secondary outcomes and adverse events, such as the number of attempts, were not tracked for this baseline cohort (Table 4).

**Table 4:** Trainee success; all intubations performed by a trainee during the study period Statistical significance was determined at *p* <0.05. **denotes Fisher’s exact test

Intubation type	Intubations Total (n)	Successful Intubations	Unsuccessful Intubations	p
All Eligible Intubations, n (%)	44	27 (61.4%)	17 (38.6%)	
Eligible Intubations; randomized to study, n (%)	31	20 (64.5%)	11 (35.5%)	0.521**
Eligible Intubations; not randomized, n (%)	13	7 (53.8%)	6 (46.2%)	
Control, n (%)	15	8 (53.3%)	7 (46.7%)	0.273**
Just In Time Training, n (%)	16	12 (75%)	4 (25.0%)	

The success by year-group was also stratified as expected based on published data, with success higher in more experienced residents.

## Discussion

This study demonstrates that the clinical implementation of just-in-time training is feasible and associated with positive outcomes. JITT rendered trainee intubations faster, more effective, and safer. JITT is simple, cost-effective, and provides an accessible, impactful way to teach, reinforce, and refine NI skills. The significance of NI skill proficiency extends beyond the delivery room to environments where neonates in acute respiratory distress may be located, such as the NICU or the emergency department. Given that NI is a high-acuity, low-volume skill, maximizing learning opportunities for each encounter is paramount. JITT and simulation have been successfully utilized to teach both routine and emergent skills in pediatrics and other medical specialties, such as emergency medicine and anesthesia [6,21]. This study provides a blueprint to improve the protocol and further enhance the validity of results in future iterations.

Although not statistically significant, the differences in overall success and first-attempt success are clinically important. The number needed to treat (NNT) for these metrics is 4.6 and 3.2, respectively (95% CI: -2,8). This indicates that the simple intervention of structured simulation targeting critical skills can improve success after only a few encounters. This is a critical point because we know that even the best simulation education loses effectiveness over time. JITT allows for an appropriate “booster” of skill training at the right time [32]. 

This investment in time and energy requires minimal expense. The Newborn Anne™ mannequin was utilized in this study based on published data, which validates its usefulness and provides objectivity in its selection. While high-fidelity trainers represent an investment, research confirms that investments in patient safety significantly decrease healthcare and hospital costs in the long run. Compared to the systemic costs of poor clinical outcomes, the investment in high-quality simulation is negligible [33,34].

Another significant finding is that procedure time was lower in both successful and unsuccessful encounters in the JITT group. One theory is that after simulation, the trainee has a clearer "experiential map" of the necessary landmarks and procedural flow. When unable to proceed as intended, the trainee, armed with realistic expectations, is more likely to abort the attempt. This awareness improves patient safety because the trainee recognizes their own discomfort or limitations, potentially sparing the infant from continued, unsuccessful intervention. This aligns with findings from international registry data, which emphasize that minimizing failed attempts is critical to improving overall safety outcomes [7].

The main strength of this study is its execution in a clinical environment, allowing for the evaluation of patient-level outcomes. While simulation research often replaces patient contact, this study demonstrates how simulation can be coupled with real-time patient encounters to provide an enhanced educational opportunity. Although not specifically tracked, trainees randomized into the JITT group reported that it was an invaluable opportunity that helped the skill “click,” providing them with increased confidence during subsequent encounters [35].

This study also recorded total procedure time stringently; any placement of a laryngoscope blade was counted as an attempt, even if suctioning was immediately required. This method provided rigorous results, as it evaluated all factors related to the intubation that affect clinical success and patient tolerance.

The primary limitation of this study is the small sample size. Despite achieving significance in several reported metrics, a larger sample is required to achieve 80% power. The study was also conducted at a single institution, which limits generalizability. However, the observed odds of success were up to 80% higher in the JITT group, which provides a strong justification for a larger future trial. Another limitation is that study personnel could not be blinded to the trainee’s randomization. While we utilized strict, objective endpoints to limit bias, a more ideal design would separate the individuals administering the intervention from those collecting the data. Nevertheless, the clinical supervisor remained blinded to the trainee’s randomization throughout the process. Finally, another limitation is lack of standardization in the pre-medication. This was beyond the scope of the study; however, emerging literature supports that pre-medication alone can increase NI success, and had this project had appropriate powering to specifically look at pre-medication, it would have been rigorously analyzed and reported on [36]. 

## Conclusions

In summary, JITT is clinically feasible in a busy NICU. Additionally, while unable to reach statistical significance, JITT improved intubations both in terms of success and safety. This study also provides an education framework for teaching this critically important skill, while also providing a theoretical framework for the teaching and maintenance of other low-volume high acuity skills (e.g., chest tube placement). 

This concept deserves continued attention and would benefit from larger and multi-site studies to achieve adequate power; however, it appears that it is a useful tool to enhance trainee education and improve patient safety.
